# GDPR-anonymous population overlap estimation

**DOI:** 10.23889/ijpds.v9i5.2711

**Published:** 2024-09-18

**Authors:** Patrick Baier

**Affiliations:** 1 Datavant

The feasibility of many collaborative, data-driven research projects in Public Health and Social Sciences depends on the size of the overlap between the populations of contributing data partners, that is, the number of individuals for whom all parties can provide data. Where the identity of respective population members is subject to privacy protections, the overlap is traditionally estimated using de-identified cryptographic tokens to represent individuals. This does however not meet the strict standard of anonymity under GDPR and potentially runs counter to emerging privacy laws of US States, and may thus impose significant regulatory and governance burdens on research institutions, even at the early scoping stage.

We present a novel method for overlap estimation, where two data partners construct a single, anonymous Bloom filter for their populations from which the overlap can be estimated. Two new techniques -- (a) the randomization of the contribution of individual population members to the Bloom filter and (b) an interactive bit-flipping protocol between data partners -- are presented which ensure that the Bloom filters are safe against dictionary attack by one party against the other party. We discuss the security aspects of this method, present the required mathematical formulas for calculating the overlap estimate, and report on the practical performance in terms of computational effort and resulting accuracy.

The goal of the method is to facilitate new research by reducing the cost and complexity of initial planning and by making the overlap estimate GDPR-anonymous, and thus safer and subject to less onerous compliance requirements.

